# Flow cytometry for the evaluation of anti-plasmodial activity of drugs on *Plasmodium falciparum *gametocytes

**DOI:** 10.1186/1475-2875-9-49

**Published:** 2010-02-11

**Authors:** Séverine Chevalley, Agnès Coste, Alexandrine Lopez, Bernard Pipy, Alexis Valentin

**Affiliations:** 1 Université de Toulouse-3, LPSNPR (Laboratoire Pharmacochimie des Substances Naturelles et Pharmacophores Redox), 118 route de Narbonne, F-31062 Toulouse cedex 9, France; 2 IRD, LPSNPR, F-31062 Toulouse, France; 3 EA2405 UMR3 MD-UM-UPS, Université Paul Sabatier Toulouse III, France

## Abstract

**Background:**

The activity of promising anti-malarial drugs against *Plasmodium *gametocytes is hard to evaluate even in vitro. This is because visual examination of stained smears, which is commonly used, is not totally convenient. In the current study, flow cytometry has been used to study the effect of established anti-malarial drugs against sexual stages obtained from W2 strain of *Plasmodium falciparum*. Gametocytes were treated for 48 h with different drug concentrations and the gametocytaemia was then determined by flow cytometry and compared with visual estimation by microscopy.

**Results and conclusions:**

Initially gametocytaemia was evaluated either using light microscopy or flow cytometry. A direct correlation (r^2 ^= 0.9986) was obtained. Two distinct peaks were observed on cytometry histograms and were attributed to gametocyte populations. The activities of established anti-malarial compounds were then measured by flow cytometry and the results were equivalent to those obtained using light microscopy. Primaquine and artemisinin had IC_50 _of 17.6 μM and 1.0 μM, respectively.

Gametocyte sex was apparently distinguishable by flow cytometry as evaluated after induction of exflagellation by xanthurenic acid. These data form the basis of further studies for developing new methods in drug discovery to decrease malaria transmission.

## Background

Many different approaches have been used to measure the susceptibility of asexual stages of *Plasmodium falciparum *to anti-malarial drugs *in vitro*. The most used methods are light microscopy, which enables visual quantification of parasitized red blood cells, and radioactive methods that evaluate the viability of parasites by tracking the incorporation of ^3^H-hypoxanthine into their nucleic acids. However, only the first method allows the quantification of gametocytes, since these cells do not multiply. Given that light microscopy is labour-intensive, subjective, and suffers from inter-operator variations, alternative methods for the counting of gametocytes are needed to increase the efficiency of drug-screening on this stage of the parasite's life-cycle.

In earlier studies, flow cytometry has been proposed to assess the viability of intra-erythrocytic stages of *Plasmodium *using DNA fluorescent stains [1,2]. Many fluorescent stains have been used in flow cytometry studies: propidium iodide [3], acridine orange [4] and YOYO-1 [5] require fixation and permeabilization of the parasites before use. Other dyes need complete lysis of erythocytes, such as Hoechst 33258 [6,7] and Picogreen^® ^[8], while hydroethine (HE) does not require lysis or fixatives. *Plasmodium *takes up and metabolizes HE into ethidium, a nucleic acid-binding fluorochrome [9]. Some authors have also proposed to combine thiazole orange with HE [10] or with Hoechst 33342 [11] to stain nucleic acids in order to differentiate intra-erythrocytic stages of *P. falciparum*.

In the present study, the discrimination, by flow cytometry, of *P. falciparum *asexual and sexual forms stained with HE was performed in order to validate a method for the screening of gametocytocidal drugs. First, the relative distribution of fluorescence in parasite subpopulations according to their maturity and sex was analyzed and then the gametocytocidal activity of drugs was determined. Finally, particular gametocyte preparations were treated with xanthurenic acid to follow the exflagellation process.

Flow cytometry was demonstrated to be usable to evaluate the different stages of sexual and asexual parasite populations and to assess the *in vitro *gametocytocidal activities of potentially anti-plasmodial drugs. Moreover, this method enables to discriminate the male and female gametocyte subpopulations.

## Methods

### Materials

Red blood cells and human serum were obtained from EFS, Toulouse (France); PBS, RPMI and additives were from Lonza (Belgium). All other reagents were from Sigma-Aldrich, l'Isle d'Abeau (France).

### *Plasmodium falciparum in vitro *culture

Parasites were cultured according to Trager and Jensen [12], and synchronized according to Lambros [13]. Briefly, parasites were routinely maintained in O+, human erythrocytes (parasitaemia: 0.5-4%, haematocrit: 4%) in RPMI 1640 with 25 mM HEPES, 2 mM L-glutamine and 7% human AB serum in a CO_2 _incubator.

Gametocyte cultures were initiated with W2-Indochina strain as described elsewhere [14], with modifications [15]. Cultures were treated with 50 mM N-acetyl-D-glucosamine for 4-5 days to remove most of the asexual stages. Old (stage IV-V, 11-13-days-old) gametocyte cultures were mostly used.

Visual counting of schizonts and gametocytes was carried out on Giemsa-stained smears before and after concentration. Images were digitally recorded with a digital camera (DS Camera Control Unit DS-U2, Nikon) mounted on an optical microscope (Nikon Eclipse 80i, Nikon).

### Purification

Magnetic purification was carried out by using the haemozoin paramagnetic complex property. Prior to purification, MACS^® ^(25LD columns, Miltenyi BioTec, Germany) columns were filled with warmed (37°C) RPMI. The experimentation was performed under sterile conditions [16,17]. The tested blood from cultures was then loaded on the columns (typically, 4 mL at 25-50% haematocrit) and warm (37°C) culture medium was then added until the eluent was apparently free of red blood cells. At this point, the columns were removed from the magnetic support after addition of 10 mL more culture medium and the eluent was recovered. It was then centrifuged (800 g, 10 min) and supernatant was discarded. The pellet was used to prepare blood smears that were Giemsa-stained and it was also analysed by flow cytometry.

### *In vitro *tests with gametocytes

Primaquine and artemisinin (the latter being kindly provided by Pierre Fabre Laboratories) were dissolved in DMSO, while chloroquine was dissolved in RPMI. Twelve day-old gametocyte cultures were transferred to 24-well or 96-well plates, and increasing dilutions of each drug were added. All experiments were performed in triplicate. After 48 h of incubation at 37°C, thin blood smears were prepared and stained with Giemsa. The number of gametocytes per 10,000 erythrocytes was visually estimated by optical microscopy. In parallel, parasitaemia was evaluated by flow cytometry.

### Flow cytometry

Cultures were washed with PBS. Pellets were resuspended in hydroethidine solution (50 μg/mL) for 20 minutes at 37°C in the dark. After washing in PBS, 10^5 ^cells were analysed with a FACScalibur cytometer (Becton Dickinson^®^) using the CellQuestPro^® ^program for data analysis.

### Confocal microscopy

In order to obtain an homogenous film, glass cover-slips were coated overnight at room temperature with poly-L-lysine (1:100 in PBS). Parasite cultures were washed twice in PBS and the pellets were resuspended in 50 μg/mL hydroethidine (HE) for 20 minutes at 37°C in the dark. Samples were then washed twice in PBS and resuspended in 1 mL of PBS. Cells were distributed on the poly-L-lysine-coated cover-slips and incubated for 20 minutes at 37°C in the dark. After cell adhesion, the cover-slips were washed with PBS and analysed with a fluorescence microscope (ConfoCor, Zeiss LM510, Carl Zeiss).

### *In vitro *exflagellation assay

The exflagellation of gametocytes was quantified according to Kawamoto *et al *[18]. Gametocytes were highly purified on MACS^® ^columns and immediately resuspended at 5% final haematocrit in 100 μM xanthurenic acid (XA) for 20 min at room temperature [19,20]. Prior to flow cytometry analysis, a visual counting of gametocytes with or without exflagellation was carried out on Giemsa-stained smears.

## Results and Discussion

**Figure 1:**
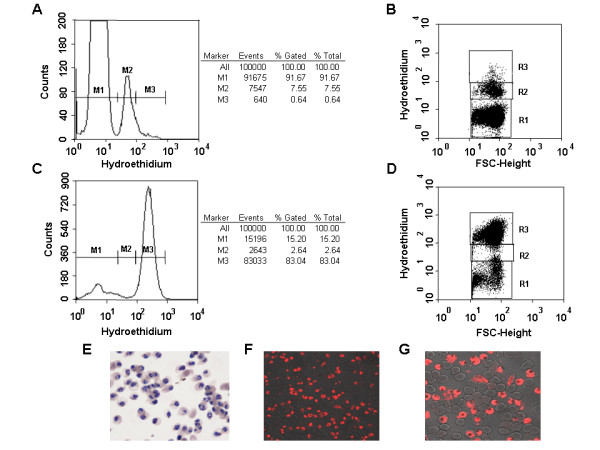
**Flow cytometry analysis of *Plasmodium falciparum *parasitized erythrocytes (W2 strain) stained with hydroethidine, asexual stages**. A and B: ring-enriched population examined by flow cytometry. C and D: schizont-enriched population: schizonts were enriched by magnetic separation through a MACS column and examined by flow cytometry. E: Giemsa-stained thin smears. F, G: HE-stained parasites (magnification × 63 (F) and zoom 2.5 (G)).

**Figure 2:**
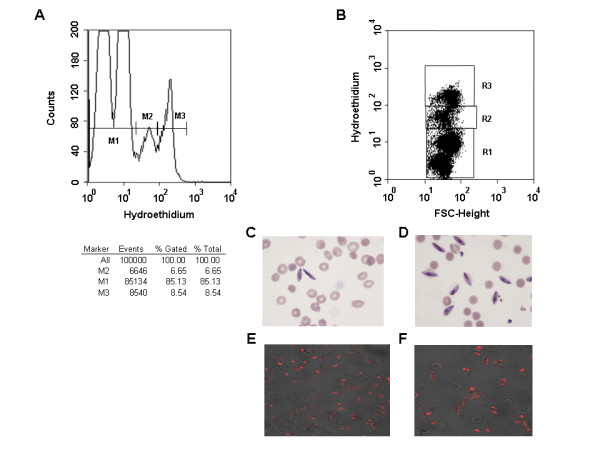
**Flow cytometry analysis of *Plasmodium falciparum *parasitized erythrocytes (W2 strain) stained with hydroethidine, gametocytes**. Gametocytes were purified by magnetic separation through a MACS column and examined by flow cytometry (A and B). Giemsa-stained thin smears (D) and HE-stained enriched culture (magnification × 63 (E) and zoom 2.5 (F)). C: Giemsa-stained thin smears before gametocyte enrichment.

*In vitro *cultures were incubated with HE, which is converted to ethidium by metabolically active cells [9]. The interaction between ethidium and parasite nucleic acids resulted in a red fluorescence emission allowing discrimination between uninfected and infected erythrocytes independently of the parasitic stage (asexual stages: Figure 1; gametocytes: Figure 2). Histograms (left panels, Figures 1A, 1C and 2A) and dot plots (right panels, Figures 1B, 1D and 2B) show the distribution of HE fluorescence (x-axis, log scale). *Plasmodium falciparum *W2 strain cultures were synchronized by 5% D-sorbitol lysis to obtain ring-enriched cultures [13], and by the magnetic enrichment method [16] in order to purify schizonts (Figure 1E) or gametocytes (Figure 2). The labelled M1 peak corresponded to non-fluorescent events (mostly uninfected erythrocytes, damaged erythrocytes and those with dead parasites inside). The fluorescence intensity of the rings was addressed to M2 peak (Figure 1A). We observed one peak M3, corresponding to late trophozoites and mature schizonts (Figure 1C). Schizonts exhibited an increase in fluorescence intensity when compared to the other stages. These results were in line with those of Pace and Staalsoe [21,22]. Flow cytometry analysis of enriched gametocytes showed two distinct peaks of fluorescence (M2 and M3) (Figure 2A). Similar results were reported in a study on Green Fluorescence Protein (GFP) fluorescence of transgenic A-SET/GFP murine *Plasmodium berghei *gametocytes [21].

Enrichment of the W2 strain at a very high yield was obtained with magnetic columns [16] with an enrichment greater than 150-fold for the schizont enriched population. For example, in one experimentation, parasitaemia (mostly late trophozoites and mature schizonts) was 0.53% before purification with MACS^® ^and 83.04% after (M3 population, Figure 1C). In another experimentation, gametocytaemia was 1.43% before and 13.7% after purification with MACS^® ^(population, Figure 2A). These results were similar to those obtained by numeration on Giemsa stained thin smears (Figures 1E, 2C and 2D). Similar results were observed throughout six independent experiments (Table 1). A true direct correlation (r^2 ^= 0.9805) was obtained between gametocytaemia determined by the two methods. More, the M2 population was, most of the times, lower than M3 (Table 1).

**Table 1:** Comparison between FCA (Flow cytometry analysis) an OM (Optical microscopy) for the determination of gametocytaemia.

		FCA	FCA	OM	OM
**Experiment number**	**M1 (%)**	**Female Gam** **(%): M2**	**Male Gam** **+ Sch (%): M3**	**Gam (%)**	**Sch (%)**

**1**	36.9	14.7	47.9	74	2.1

**2**	66.9	2.5	28.7	38	19

**3**	85.1	6.6	8.5	18.9	0.5

**4**	80.9	12.6	6.4	13.4	0

**5**	83.3	3.5	13.3	17	0

**6**	90.9	5.8	2.5	6.9	1

**7**	93.6	1.6	4.6	3	1

**mean**	76.8	6.8	16.0	24.4	3.3

Parasitaemias of *in vitro P. falciparum *gametocyte cultures (W2 strain) were evaluated by flow cytometry and optical microscopy (FCA: flow cytometry assay; OM: optical microscopy). These values correspond to the percentages of every population after gametocyte (Gam) enrichment by MACS.

M1: percentage corresponding to the first peak of the histogram in FCA, which corresponds to non-fluorescent events (uninfected erythrocytes).

M2: parasitaemia corresponding to the second peak of the histogram in FCA (erythrocytes infected by gametocytes).

M3: parasitaemia corresponding to the third peak of the histogram in FCA (erythrocytes infected by gametocytes and/or residual asexual stages (schizonts: Sch)).

**Figure 3:**
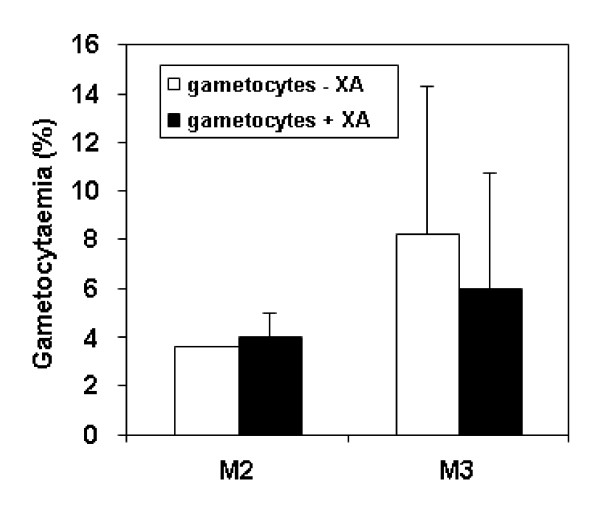
**Effect of xanthurenic acid (XA) on gametocytaemia evaluated by flow cytometry**. Experiments were performed on MACS^® ^enriched gametocyte culture. Percentages of each population (M2 and M3) of a gametocyte culture before or after exflagellation with 100 μM xanthurenic acid (XA) were evaluated by flow cytometry (mean data of three independent experiments ± SD, the parasitaemia level varied highly between these three experiments). As no decrease of gametocytaemia was observed in M2 population after XA treatment, this group represented potentially female gametocytes. The decrease observed in the M3 population after XA treatment corresponded to mature male gametocytes exflagellation. The residual M3 population after XA treatment was mostly immature gametocytes and schizonts.

The repartition of gametocytes in each population (M2 and M3) could be due to the sex of the gametocytes, which modifies their amount of DNA, and/or to their stage of maturation. To determine whether the M2 and M3 peaks corresponded to the difference between male and female gametocytes, we compared gametocyte cultures before and after male gametogenesis. Exflagellation has been achieved by decreasing drastically the culture temperature (from 37°C to 23°C) and submitting the gametocytes to xanthurenic acid (XA), a gametocyte-activating factor (GAF) [23,24]. The sensitivity of *P. falciparum *microgametocytes to XA was determined *in vitro *[19] and the exflagellation response was expressed as a percentage of a 100 μM XA control. The effector concentration for half-maximal response (EC_50_) in cultured *P. falciparum *NF54 strain was 2 μM. In the current study, after enrichment by MACS^®^, a fraction of the purified gametocytes (strain W2) was treated with 100 μM XA, a concentration that triggers the highest stimulatory effect on *in vitro *exflagellation [19]. This XA-stimulated culture was compared with the non-stimulated gametocytes by flow cytometry. The data are presented in Figure 3. Firstly, a slight increase of gametocytaemia was observed in the M2 population. Secondly, the exflagellation by the addition of XA decreased the gametocytaemia only in the M3 peak. The large error observed (Figure 3) can be attributed to the variability in the parasitaemia levels observed in the three independent experiments represented. Altogether these data showed that the decrease in the M3 peak after XA corresponded to a decrease in male gametocytes and hence showed that in the M2 population mostly female ones were present, unlike M3 population that contained mature male gametocytes and residual schizonts (Table 1). On one hand, the observation that the M2 population was less fluorescent (one female gametocyte leads to a single gamete) than the M3 populations, including numerous male gametocytes which leads to the formation of eight gametes was in agreement with the study by Pace *et al *[21]. These authors demonstrated that GFP fluorescence of late female gametocytes, identified by the sex-specific antibody Pfg377, was less intense than the fluorescence generated by male gametocytes [21]. On the other hand, the percentage of female gametocytes was higher than the percentage of mature male ones (Figure 3). These results were in accordance with sex ratio studies in *P. falciparum *that have showed that female gametocytes were generally more numerous than males. Generally, one male was observed for four females, though large fluctuations in the gametocyte sex ratio have been observed [25].

To validate the use of flow cytometry in order to evaluate the *in vitro *gametocytocidal activity of drugs, the efficiency of primaquine, artemisinin and chloroquine against gametocytes were compared both by the classic optical microscopy (OM) method and by FCA. After 12 days of maturation and treatment with N-acetyl-D-glucosamine (5 days before testing) to remove most of asexual stages, gametocytes obtained from chloroquine-resistant W2 strain were exposed to these established anti-malarial compounds at increasing dilutions for 48 h. The results are summarized in Table 2. As expected, artemisinin and primaquine proved to be transmission-blocking drugs (artemisinin IC_50 _against gametocytes was 1,000 nM by FCA and 690 nM by OM). As expected, chloroquine was inactive (IC_50 _> 20,000 nM by FCA and by OM). Primaquine, which had an IC_50 _against gametocytes of 17,600 nM by FCA and 11,200 nM by OM could be compared with gametocytaemia evaluated by OM by Sall *et al *(namely 9600 μM [26]). Interestingly, the results obtained with the two methods (OM and FCA) were similar for the three anti-malarial compounds. Therefore, drug effects on gametocytaemia, which are usually analysed by conventional microscopy, could clearly be assessed by the flow cytometric method.

**Table 2:** Conventional drugs activity against *P. falciparum *gametocytes.

	FCA	OM
Primaquine	17.60	11.20

Artemisinin	1.00	0.69

Chloroquine	> 20	> 20

*In vitro *anti-malarial activity of primaquine, artemisinin and chloroquine against *P. falciparum *gametocytes (W2 strain) evaluated by flow cytometry and optical microscopy (FCA: flow cytometry assay; OM: optical microscopy) (means of the data of three experiments). Values are given in μM.

## Conclusions

FCA, in combination with magnetic enrichment, has here been shown to be useful to estimate the inhibitory concentrations of known drugs against *P. falciparum *gametocytes and hence should be useful to evaluate promising anti-gametocyte drugs. Moreover, HE-labelled viable parasites, which were the only labelled cells, while Giemsa staining did not allow the differentiation between living and dead parasites. Although these results were obtained on *P. falciparum*, they are close to those of other authors [21,27] who have quantified GFP expression during intraerythrocytic development of transgenic A-SET/GFP *P. berghei*. They showed that the fluorescence intensity of gametocytes was comparable to that of late trophozoites, as showed in the present report. Using flow cytometry, other researchers have identified *P. falciparum *gametocytes well before they were morphologically distinguishable from asexual stage parasites, thanks to the use of the chimeric Pfs 16-GFP [28]. This FACS based assay used one of the earliest known gametocyte proteins, the Pfs16 as a reporter. However, the present method can be used for non-genetically modified parasites.

## Competing interests

The authors declare that they have no competing interests.

## Authors' contributions

SC conceived and carried out the studies, drafted the manuscript. AC conceived the study, interpreted cytometry studies, redaction and correction of the manuscript. AL master degree student in the laboratory, participated to in vitro culture and cytometry studies. BP interpreted cytometry studies, drafted and corrected the manuscript. AV conceived the study and participated in its design and coordination, redaction and correction of the manuscript.

All authors read and approved the final manuscript.
